# Soluble CD157 in pleural effusions: a complementary tool for the diagnosis of malignant mesothelioma

**DOI:** 10.18632/oncotarget.25237

**Published:** 2018-04-27

**Authors:** Stefania Augeri, Stefania Capano, Simona Morone, Giulia Fissolo, Alice Giacomino, Silvia Peola, Zahida Drace, Ida Rapa, Silvia Novello, Marco Volante, Luisella Righi, Enza Ferrero, Erika Ortolan, Ada Funaro

**Affiliations:** ^1^ Laboratory of Immunogenetics, Department of Medical Sciences, University of Torino, Torino 10126, Italy; ^2^ Department of Oncology, University of Torino, San Luigi Hospital, Torino 10043, Italy

**Keywords:** CD157/Bst1, mesothelioma, biomarker, pleural effusion, diagnosis

## Abstract

**Background:**

CD157/Bst1 glycoprotein is expressed in >85% of malignant pleural mesotheliomas and is a marker of enhanced tumor aggressiveness.

**Results:**

*In vitro*, mesothelial cells (malignant and non-malignant) released CD157 in soluble form or as an exosomal protein. *In vivo*, sCD157 is released and can be measured in pleural effusions by ELISA. Significantly higher levels of effusion sCD157 were detected in patients with malignant pleural mesothelioma than in patients with non-mesothelioma tumors or with non-malignant conditions. In our patient cohort, the area under the receiver-operating characteristic curve for sCD157 that discriminated malignant pleural mesothelioma from all other causes of pleural effusion was 0.685, cut-off (determined by the Youden Index) = 23.66 ng/ml (62.3% sensitivity; 73.93% specificity). Using a cut-off that yielded 95.58% specificity, measurement of sCD157 in cytology-negative effusions increased sensitivity of malignant pleural mesothelioma diagnosis from 34.42% to 49.18%.

**Conclusions:**

Evaluation of soluble CD157 in pleural effusions provides a diagnostic aid in malignant mesothelioma.

**Methods:**

Soluble CD157 (sCD157) was detected biochemically in culture supernatants of malignant and non-malignant mesothelial cells, and in pleural effusions from various pathological conditions. An ELISA system was established to measure the concentration of sCD157 in fluids, and extended to analyze sCD157 in pleural effusions from a cohort of 295 patients.

## INTRODUCTION

Malignant pleural mesothelioma (MPM) is an incurable tumor that originates from mesothelial cells lining the pleural cavity. MPM is associated with asbestos exposure and because of its long latency, tumor incidence is predicted to increase significantly in the next decade, especially in countries where asbestos has not been banned [1]. Although Italy banned asbestos in 1992, mesothelioma remains a major public health concern due to work and general environmental exposure to asbestos, especially in the industrial north. Due to its insidious onset and delay in clinical detection, MPM is typically diagnosed at advanced stages and the overall prognosis is poor [2]. Palliative combined platinum and anti-folates chemotherapy is considered standard care but gives a modest survival advantage of approximately three months, compared to cisplatin alone [3].

Over 80% of MPM patients present dyspnea with malignant pleural effusion [4], caused in part by increased vascular permeability and tissue leakage. Pleural fluid obtained by thoracentesis often contains malignant cells, and effusion cytology constitutes the most common method for establishing a diagnosis. However, in MPM, the sensitivity of cytological evaluation is low thus histological examination of biopsy specimens obtained by thoracoscopy is considered the gold standard diagnostic procedure [5, 6].

This dismal clinical scenario highlights the urgent need for more sensitive and non-invasive tools to aid the diagnostic workflow. In this setting, tumor biomarkers can play a meaningful role in diagnosis and prognosis, in predicting and monitoring treatment responses, and in screening for early detection of disease [7].

CD157/Bst1 is a cell-adhesion glycoprotein encoded by the human bone marrow stromal cell 1 (*BST1*) gene, which maps to 4p15.32 [8] and belongs to the ADP-ribosyl cyclase gene family [9, 10]. In addition to the canonical protein of 318 amino acids, a second CD157 proteoform of 333 amino acids has been recently identified [11]. The two proteins share similar distribution and functions, but only the canonical form displays NAD glycohydrolase activity. CD157 exists both as glycosylphosphatidylinositol (GPI)-anchored membrane protein implicated in the control of leukocyte trafficking [12], and as a soluble protein whose biological role is as yet unknown [13]. CD157 is highly expressed in monocytes, polymorphonuclear leukocytes (PMN) [14] and in more immature myeloid stages [15], and also in vascular endothelial cells [16] and bone marrow stromal cells [17]. CD157 is also expressed in 97% of acute myeloid leukemia patients and is currently under investigation as a potential candidate for antibody-based targeted therapy [18]. CD157 regulates cell adhesion and migration by high affinity binding to selected components of the extracellular matrix within their heparin binding domains [19].

Over the past decade, we have established a link between CD157 expression and prognosis in certain solid tumors [20, 21]. CD157 is expressed in >90% of epithelial ovarian cancers where high CD157 expression correlates with increased tumor aggressiveness, promotes epithelial-to-mesenchymal transition [22] and is an independent prognostic factor for overall survival [23]. CD157 is also expressed in >85% of MPM, and again, high CD157 expression is associated with enhanced tumor aggressiveness and with reduced sensitivity to platinum-based chemotherapy, notably in the biphasic histotype [24]. The combination of CD157 expression in MPM and the knowledge that a soluble form of the protein has been detected in serum of patients with autoimmune disorders [13] suggested we examine pleural fluid in MPM for the presence of soluble CD157 (sCD157). In this study, first we explored the ability of MPM cells to produce sCD157 *in vitro*, and then we investigated the presence of sCD157 in pleural effusions. Finally, we performed a first assessment of the potential clinical utility of sCD157 in MPM patients.

## RESULTS

### Pleural mesothelium-derived cell lines release CD157 in cell culture supernatants

**Figure 1:**
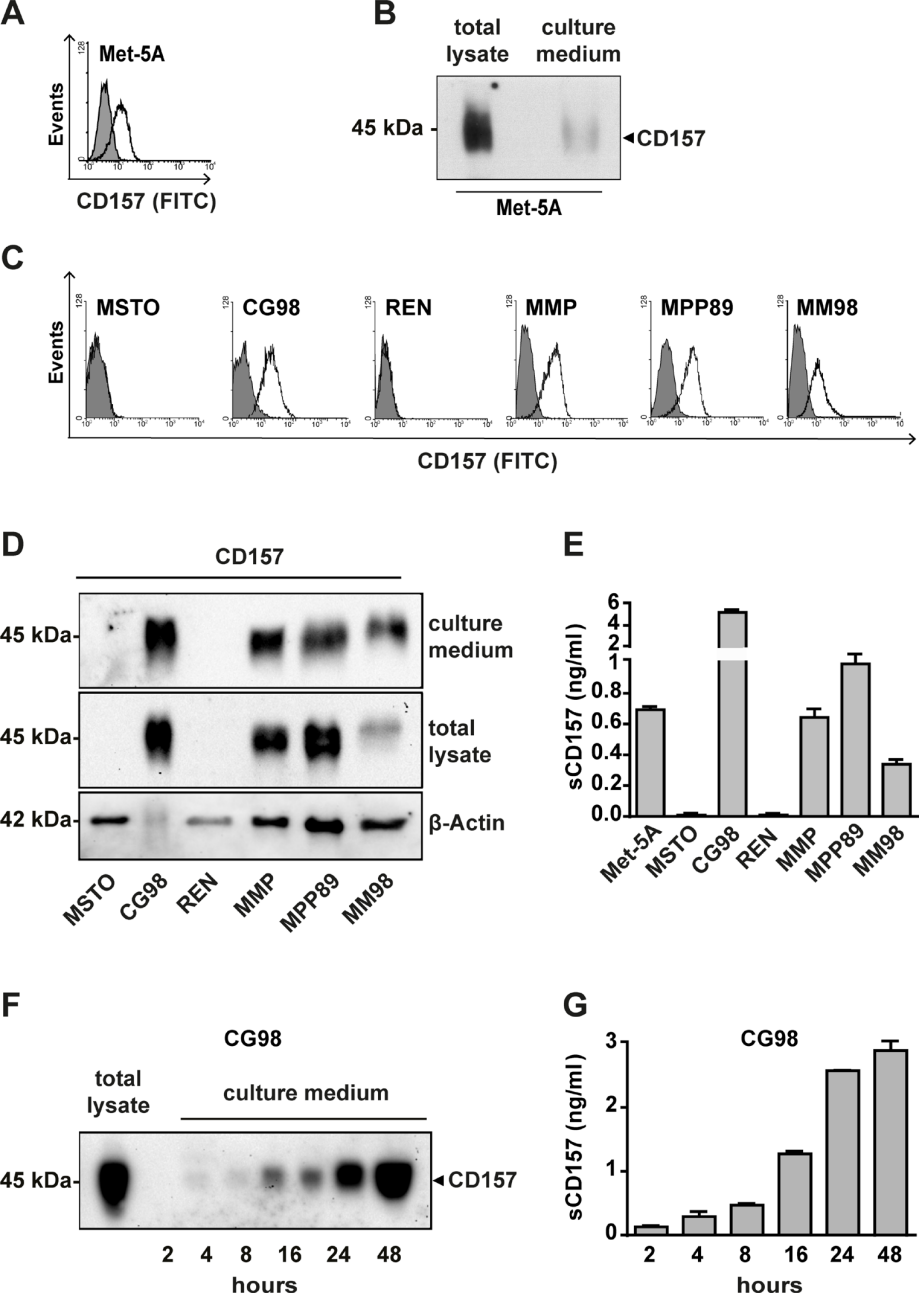
Analysis of CD157 expression and release in malignant and non-malignant mesothelial cell lines Flow cytometric analysis of CD157 expression in (**A**) Met-5A non-malignant mesothelial cells and (**C**) six MPM cell lines. In each sample, 10^4^ cells were analysed; x-axis = fluorescence intensity, y-axis = number of cells (events). White histograms represent the expression of membrane CD157 in live cells analysed by the SY/11B5 mAb, shaded histograms indicate isotype-matched control mAb. Western blot analysis of CD157 in (**B**) Met-5A cells and (**D**) six MPM cell lines maintained in serum-free culture medium for 48 hours. Proteins were separated by SDS-PAGE in non-reducing conditions, blotted and probed with the SY/11B5 anti-CD157 mAb; β-Actin was used as a loading control of the total lysates. One representative experiment is shown (*n* = 3). (**E**) sCD157 was measured by the double determinant ELISA described in Materials and Methods, in serum-free culture medium of the indicated cell lines. Results are expressed as ng/ml of soluble CD157 and histograms represent the mean ± s.e.m. of three experiments performed in duplicate. (**F**) Western blot time-course analysis of sCD157 accumulated in serum-free culture medium of CG98 MPM cells. Proteins were precipitated by TCA, separated by SDS-PAGE in non-reducing conditions, transferred to PVDF membrane and probed with the SY/11B5 anti-CD157 mAb. One representative experiment is shown (*n* = 2). (**G**) Quantification of sCD157 concentration by the double-determinant ELISA in serum-free culture medium of CG98 MPM cells at the indicated time points. Results are expressed as ng/ml of soluble CD157. Histograms represent the mean value ± s.e.m. of three experiments performed in duplicate.

To assess the ability of cells of mesothelial origin to shed CD157 in culture supernatants, we selected the following cell lines: (i) Met-5A, a non-malignant mesothelial cell line, CD157-positive; (ii) CG98, MMP, MPP89, and MM98 mesothelioma, CD157-positive; and (iii) MSTO-211H and REN mesothelioma, CD157-negative cell lines. The CD157 expression status for each cell line was confirmed by flow cytometry (Figure 1A, 1C).

Cells were maintained in serum-free medium for 48 hours, then culture media and cells were collected separately for western blot analysis. CD157 was detected in supernatants from Met-5A cells (Figure 1B) and from the CD157-positive MPM cell lines (but not in supernatants from the two CD157-negative MPM cell lines) (Figure 1D). By western blotting, we observed the typical 45–50 kDa smear, a characteristic feature shared with membrane-bound CD157 (Figure 1D) due to heterogeneous glycosylation [25]. Thus, soluble forms of CD157 are released *in vitro* by non-malignant and malignant mesothelial cells, and these forms are recognized by the same SY/11B5 mAb that detects membrane-bound CD157.

Next, we set up a double determinant ELISA to measure the concentration of sCD157 in culture supernatants from our mesothelial cell panel. The assay confirmed that all CD157-positive cells released sCD157 into the culture medium, in variable amounts depending on the cell line. As expected, sCD157 was not detected in supernatants from CD157-negative cells (MSTO-211H and REN) (Figure 1E). As CG98 MPM cells released the most sCD157, they were used to investigate the kinetics of sCD157 accumulation *in vitro*. Using sub-confluent CG98 cell culture medium, sCD157 was detected within 2 hours of serum removal, and continued to increase up to 48 hours (Figure 1F, 1G). Beyond this timeframe, the viability of serum-deprived cells was compromised, preventing further analysis.

**Figure 2:**
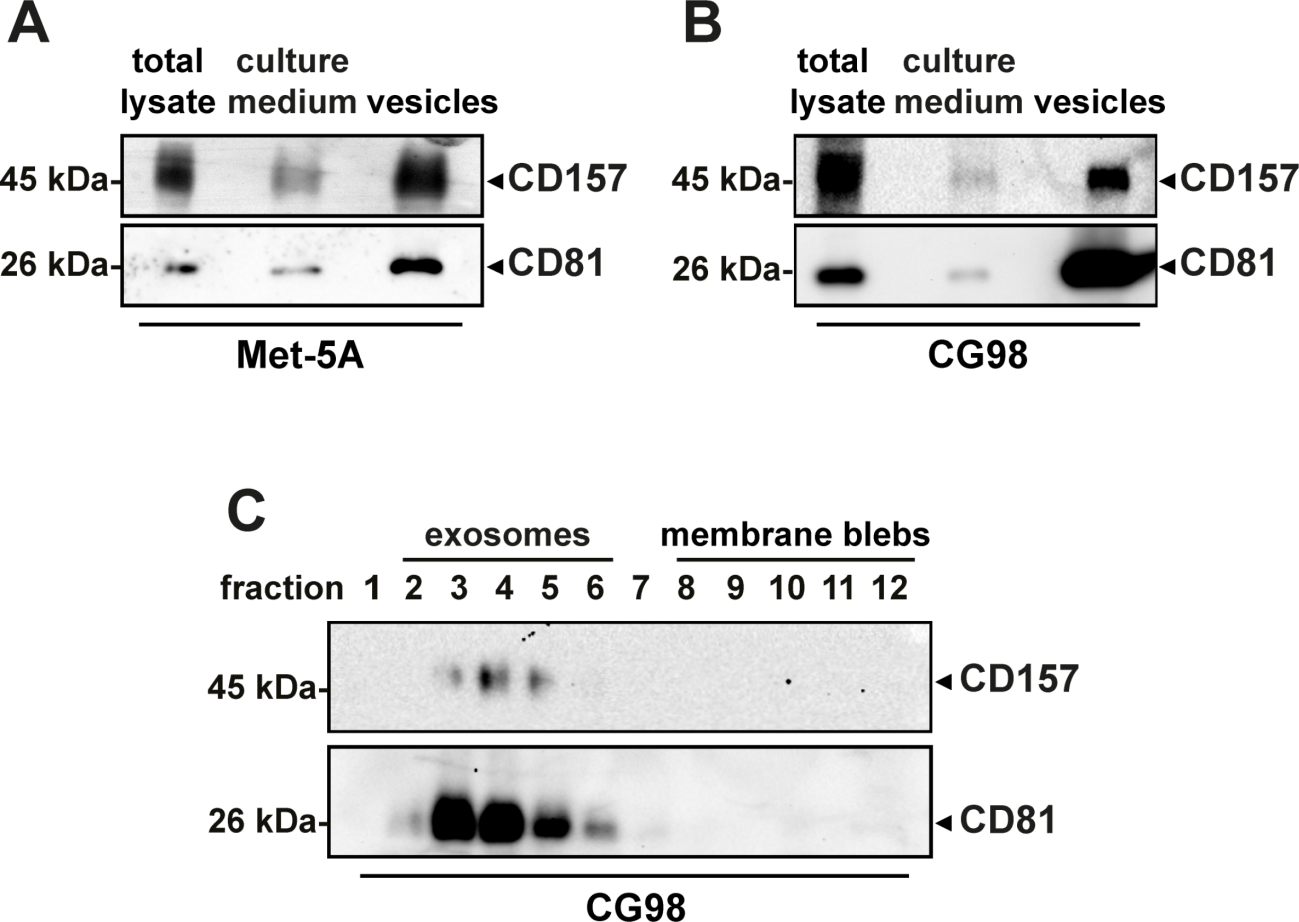
Analysis of CD157 expression in vesicles released by mesothelial cells Western blot analysis of CD157 expressed in total cell lysate (30 μg/lane), cell culture medium (1 ml, TCA precipitated) and vesicles (30 μg/lane) from (**A**) non-malignant Met-5A mesothelial cells or (**B**) CG98 mesothelioma cells, detected by SY/11B5 mAb. A representative experiment is shown (*n* = 3). (**C**) Western blot analysis of CD157 and CD81 expression in exosomes obtained from serum-free culture medium of CG98 cells by sucrose density gradient fractionation. Twelve fractions were collected from the top of the gradient, proteins from each fraction were precipitated with methanol/chloroform, separated by 10% SDS-PAGE, transferred to PVDF membranes and immunoblotted with anti-CD157 or anti-CD81 mAb. A representative experiment is shown (*n* = 3).

GPI-anchored CD157 is found in detergent-resistant microdomains [26] and may be secreted via exosomes [27, 28]. To assess this possibility, culture medium was obtained from Met-5A (mesothelial cells, CD157-positive) and CG98 (MPM, CD157-positive) and ultracentrifuged to obtain the microvesicle pellet. By western blotting, we detected CD157 in the pellet from both Met-5A and CG98 cell supernatants (Figure 2A, 2B). Sucrose density gradient fractionation of the CG98 microvesicle pellet then showed that CD157-positive vesicles were included in the low-density sucrose fractions enriched in exosomes (Figure 2C), as confirmed by coexpression of CD81, a tetraspanin protein considered a prototypic exosome marker [29, 30]. These findings demonstrate that, *in vitro*, CD157 is at least partly released in the form of exosome-bound protein both by benign and malignant mesothelial cells.

### Identification of CD157 in pleural effusions

**Figure 3:**
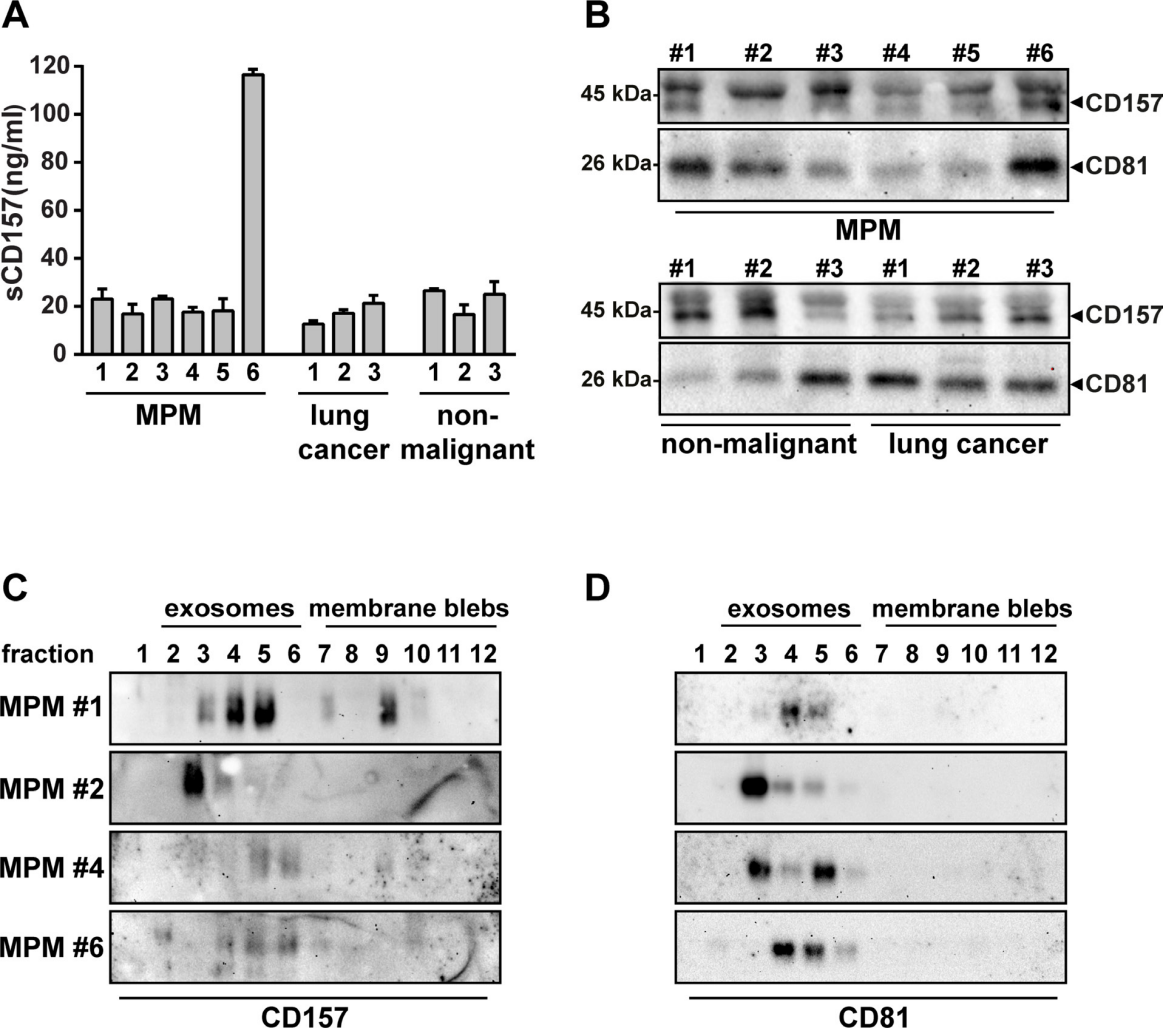
Identification of soluble CD157 in pleural effusions (**A**) Detection of sCD157 in pleural effusions from patients with different malignant and non-malignant thoracic diseases, measured by the double determinant ELISA. Results are expressed as ng/ml of soluble CD157, and histograms are the mean value ± s.e.m. of three independent experiments performed in duplicate. (**B**) Representative western blot analysis of CD157 and CD81 expressed by exosomes purified using the Total Exosomes Isolation kit from 200 μl of pleural effusion from patients with MPM (6), lung cancer (3) or non-malignant thoracic pathologies (3). Exosome preparations were separated by SDS-PAGE, transferred to PVDF membrane and probed with anti-CD157 or anti-CD81 mAb. (**C**) and (**D**) Vesicles were purified by ultracentrifugation of pleural effusions from four MPM patients and subjected to sucrose density gradient fractionation. Twelve fractions were collected from the top of the gradient, proteins from each fraction were precipitated with methanol/chloroform, separated by 10% SDS-PAGE, transferred to PVDF membranes and immunoblotted with (**C**) anti-CD157 or (**D**) anti-CD81 mAb. One representative experiment is shown (*n* = 2).

We then investigated the possible release of sCD157 by mesothelial cells *in vivo* and its eventual presence in pleural effusions. There are many causes of fluid build-up in the pleural cavity, from lung infections, heart failure, to primary and metastatic cancers. Therefore, we measured the sCD157 content in twelve representative pleural effusions of diverse pathological origin: 6 MPM, 3 lung cancer and 3 non-cancerous (1 pleurisy, 2 chronic inflammation). Soluble CD157 was detected by ELISA in all 12 effusion samples, in varying concentrations (Figure 3A), confirming that sCD157 is released and measurable in pleural effusions.

Effusions were also analysed to establish if we were also detecting CD157-positive exosomes. Exosomes were isolated from the same 12 effusions analysed above, exploiting a procedure (Total Exosome Isolation Kit) that efficiently isolates intact exosomes from small amounts of effusion [31]. Western blot analysis detected both CD157 and the exosome marker CD81 in all effusions (Figure 3B). We then focused on exosomes derived from the MPM patients, and had sufficient amounts of pleural effusion from 4 of the 6 MPM samples (nos. 1, 2, 4 and 6) for sucrose density gradient fractionation which confirmed the presence of CD157-positive MPM exosomes. Indeed, in the 4 samples, sCD157 was mainly detected in the low-density sucrose fractions (Figure 3C) containing the CD81-positive exosomes (Figure 3D).

### Quantification of soluble CD157 levels in pleural effusions

Next, to investigate the potential clinical utility of sCD157 as a biomarker in MPM patients, we retrospectively measured sCD157 concentration in pleural effusions obtained from 295 consecutive patients of whom 61 (20.67%, 40 male/21 female) were diagnosed with MPM, 129 had non-MPM malignancies, and 105 had effusions of non-neoplastic origin (Table 1). For 21 of the MPM patients, the diagnosis was based on effusion cytology and confirmed by immunohistochemistry of biopsy specimen. For 33 MPM, effusion cytology was inconclusive so diagnosis was made by immunohistochemistry of biopsy specimens. For 7 MPM patients, thoracoscopy was not possible and diagnosis was based on effusion cytology with support from the clinicopathological and radiographic findings. For the latter, the histological subtype could not be defined whereas among the other MPM, 47 (77.05%) were epithelioid, two (3.28%) were biphasic and five were (8.2%) sarcomatoid. Of the 129 patients with non-MPM malignancy (43.73%, 76 male/53 female), 82 had lung cancer and 47 had cancer of non-pulmonary origin. In 105 patients (35.6%, 66 male/39 female) effusions were due to non-malignant thoracic disorders, including pleurisy, chronic inflammation, heart failure, trauma, tuberculosis, pneumothorax and effusions of unspecified origin. There was no significant difference in age between the groups (for patient characteristics, see Table 1).

**Table 1:** Patients characteristics and sCD157 levels in pleural effusions in each group

Diagnosis	Gender (M/F)	Median age, years (range)	Number of cases (%)	sCD157 (ng/ml) median (IQR)	*P* value^**^
**Pleural effusions (*n* = 295)**					
**MPM (*n* = 61)**	40/21	76 (52–94)		**31.02 (17.97–51.51) ^ref^**	
Epithelial			47 (77.05)		
Biphasic			2 (3.28)		
Sarcomatoid			5 (8.20)		
NOS^*^			7 (11.48)		
**Non-MPM metastatic cancers (*n* = 129)**					
**Lung cancer (*n* = 82)**	56/26	73 (33–93)		**18.71 (11.12–23.7)**	<0.0001
Non-small cell			16 (19.51)		
Adenocarcinoma			42 (51.22)		
Squamous cell			4 (4.88)		
Unknown histotype			20 (24.34)		
**Other cancers (*n* = 47)**	20/27	76 (20–94)		**19.8 (10.53–31.25)**	0.0049
Breast			11 (23.40)		
Lymphoma			8 (17.02)		
Colorectal			5 (10.64)		
Leukemia			4 (8.51)		
Ovary			3 (6.38)		
Renal			3 (6.38)		
Hepatocellular			2 (4.26)		
Pleural			2 (4.26)		
Gastric			1 (2.13)		
Pancreatic			1 (2.13)		
Sarcoma			1 (2.13)		
Peritoneum			1 (2.13)		
Unknown primary			5 (10.64)		
**Non-malignant diseases (*n* = 105)**	66/39	79 (24–95)		**18.6 (11.70-24.10)**	<0.0001

^*^Not otherwise specified.

^**^P value between indicated groups and the MPM group as the reference^(ref)^.

**Figure 4:**
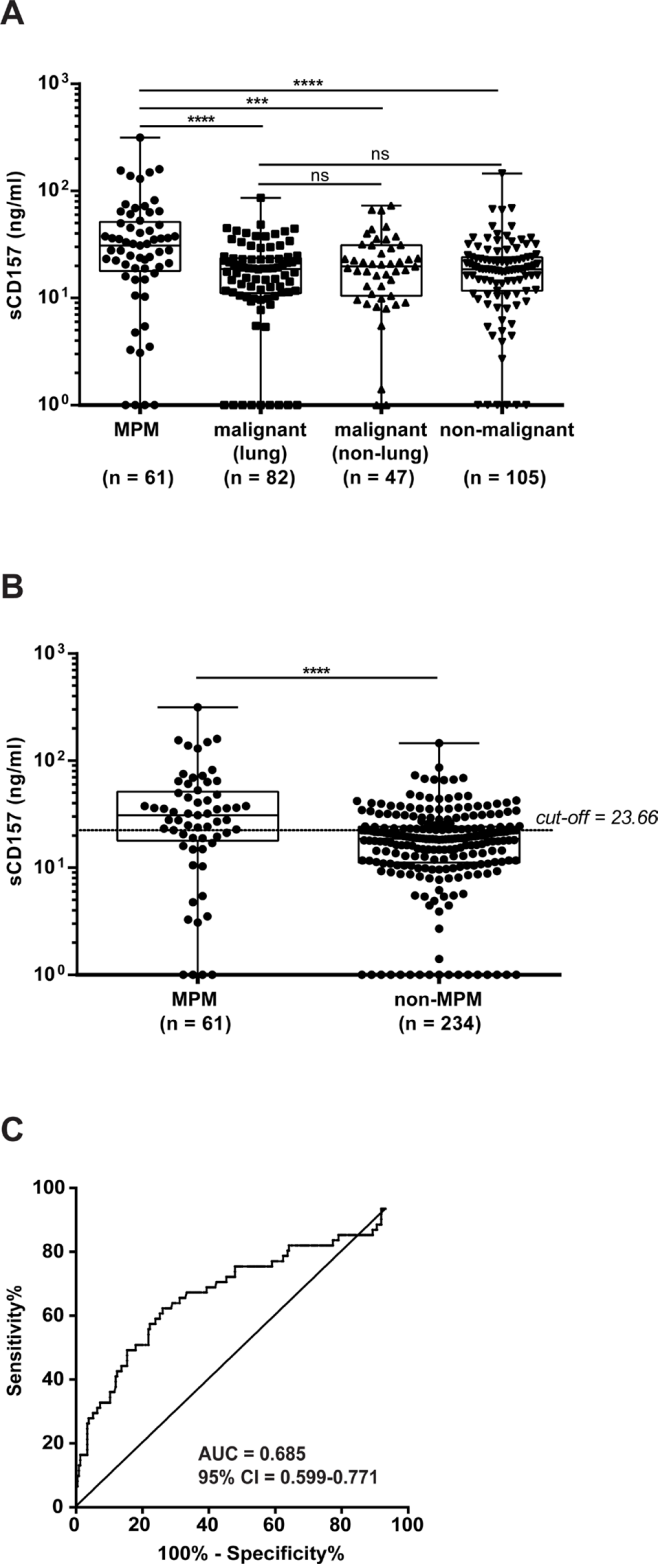
Quantification of sCD157 levels in pleural effusions sCD157 was measured by the double determinant ELISA in 295 pleural effusions, and the levels of sCD157 in pleural effusions from patients with MPM (*n* = 61) were compared with (**A**) other metastatic cancers of lung (*n* = 82) or non-lung (*n* = 47) origin, or with non-malignant pathologies (*n* = 105). (**B**) sCD157 levels in pleural effusions from patients with MPM (*n* = 61) were compared with all pleural effusions from patients with non-MPM conditions (*n* = 234). Each data point corresponds to sCD157 concentration of a single effusion sample, and represents the mean value of three independent experiments performed in duplicate. Boxes indicate the range (25th-75th percentiles), whiskers indicate major and minor values; the horizontal line within the boxes indicates the median sCD157 concentration of each group. (Mann–Whitney *U*-test, ^****^P < 0.0001; ^***^P = 0.00049; ns = not significant). (**C**) The optimal cut-off for discriminating MPM from all other thoracic diseases was determined by receiver operating characteristic (ROC) curve analyses of the area under the ROC curve (AUC) for MPM patients versus non-MPM patients (AUC = 0.685; 95% CI = 0.599–0.771, sensitivity = 62.3% and specificity = 73.93%). Horizontal dashed line indicates the cut-off determined by the Youden Index (sCD157 = 23.66 ng/ml).

Soluble CD157 was measured in all 295 pleural effusions. MPM effusions had significantly higher concentrations of sCD157 than those from patients with either non-MPM malignancies [regardless of their lung (*P* < 0.0001) or non-lung origin (*P* = 0.00049)] or non-malignant diseases (*P* < 0.0001). The effusion concentration of sCD157 in patients with non-MPM malignancies or with non-malignant diseases were comparable (Figure 4A) (Table 1).

When we compared MPM effusions (61) versus all non-MPM effusions (234), sCD157 concentration was significantly higher in MPM patients (*P* < 0.0001) (Figure 4B) and not influenced by patient age or gender (*data not shown*). A ROC curve was generated to assess the ability of sCD157 concentration in pleural effusions to distinguish between MPM patients and all other patients in this study: the AUC for sCD157 was 0.685 (95% CI = 0.599–0.771) (Figure 4C). Using the maximum value of the Youden Index, the sCD157 cut-off point was 23.66 ng/ml. At a threshold of 23.66 ng/ml, sCD157 sensitivity was 62.3% and specificity was 73.93%.

In further ROC analyses, MPM effusions yielded an AUC of 0.697 (95% CI = 0.606–0.789; *P* < 0.0001) versus lung cancer effusions, an AUC of 0.657 (95% CI = 0.554–0.761; *P* = 0.0052) versus non-lung cancer effusions, and an AUC of 0.688 (95% CI = 0.596–0.779; *P* < 0.0001) versus non-malignant effusions. In conclusion, sCD157 levels were significantly higher in MPM effusions than in other types of pleural effusion. However, sCD157 alone does not reach sufficient sensitivity and specificity as diagnostic marker to discriminate all patients with MPM from the other patient groups.

### Soluble CD157 aids cytological diagnosis of MPM

Although not diagnostic *per se*, sCD157 could contribute to the cytological diagnosis of MPM. To explore this possibility, the 75th percentile (sCD157 = 51.51 ng/ml) was chosen as cut-off point to minimize the probability of a false-positive result. This cut-off yielded 96.58% specificity for MPM effusions versus all other effusions, regardless of their origin. In order to compare sCD157 concentration with the cytological diagnosis performed on all 295 pleural effusions, the cytopathology reports were reviewed. These revealed that 162 (55%) pleural effusions were classified as cytology-negative, 38 (13%) as suspicious of malignancy or containing atypical mesothelial cells, 74 (25%) were diagnosed as non-MPM tumors, and 21 (7%) as MPM.

**Figure 5:**
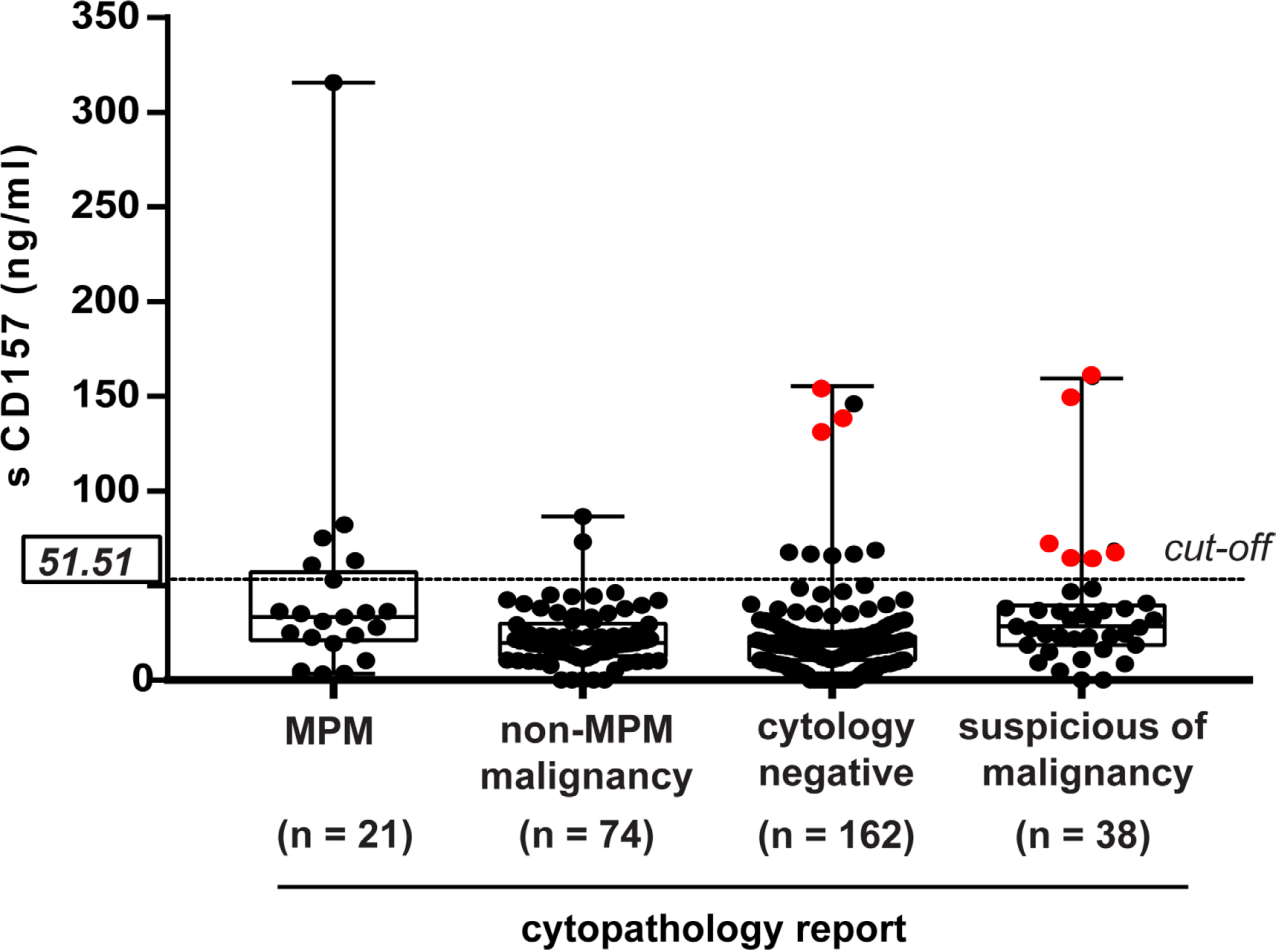
Contribution of sCD157 to the cytological diagnosis of MPM sCD157 concentrations in pleural effusions were plotted against the diagnosis established by cytopathology. Each data point indicates mean concentration of sCD157 of a single effusion sample and represents the mean value of three independent experiments performed in duplicate. Horizontal dashed line indicates the cut-off corresponding to the 75th percentile (sCD157 = 51.51 ng/ml). Boxes indicate the range (25th–75th percentiles), whiskers indicate major and minor values; the horizontal line within the boxes indicates the median sCD157 concentration of each group. Red dots highlight nine effusions from MPM patients with sCD157 ≥51.51 ng/ml, not diagnosed by cytology.

In the cytology-negative group, high sCD157 (≥51.51 ng/ml) was detected in 9/162 (5.55%) effusions. Of this cytology-negative/high sCD157 group: 3/9 were diagnosed as MPM based on the biopsy specimens (Figure 5); 2/9 were patients with a previous diagnosis of cancer (Hodgkin's lymphoma, sarcomatoid kidney carcinoma), and 4/9 were from patients with non-malignant diseases [pleurisy (1), tuberculosis (1) and chronic inflammation with elevated PMN (2)]. Of 38 pleural effusions classified as suspicious of malignancy, 6 (15.79%) had sCD157 levels ≥51.51 ng/ml, and all 6 were effusions from MPM patients (Figure 5). In conclusion, by using the same pleural effusion sample to measure sCD157 and to evaluate cytology, we were able to make a diagnosis of mesothelioma in 30/61 MPM patients in our cohort, raising the sensitivity of effusion-based MPM diagnosis from 34.42% (cytology alone) to 49.18% (cytology + sCD157).

### Analysis of MPM patients with high and low sCD157 levels in pleural effusion

**Figure 6:**
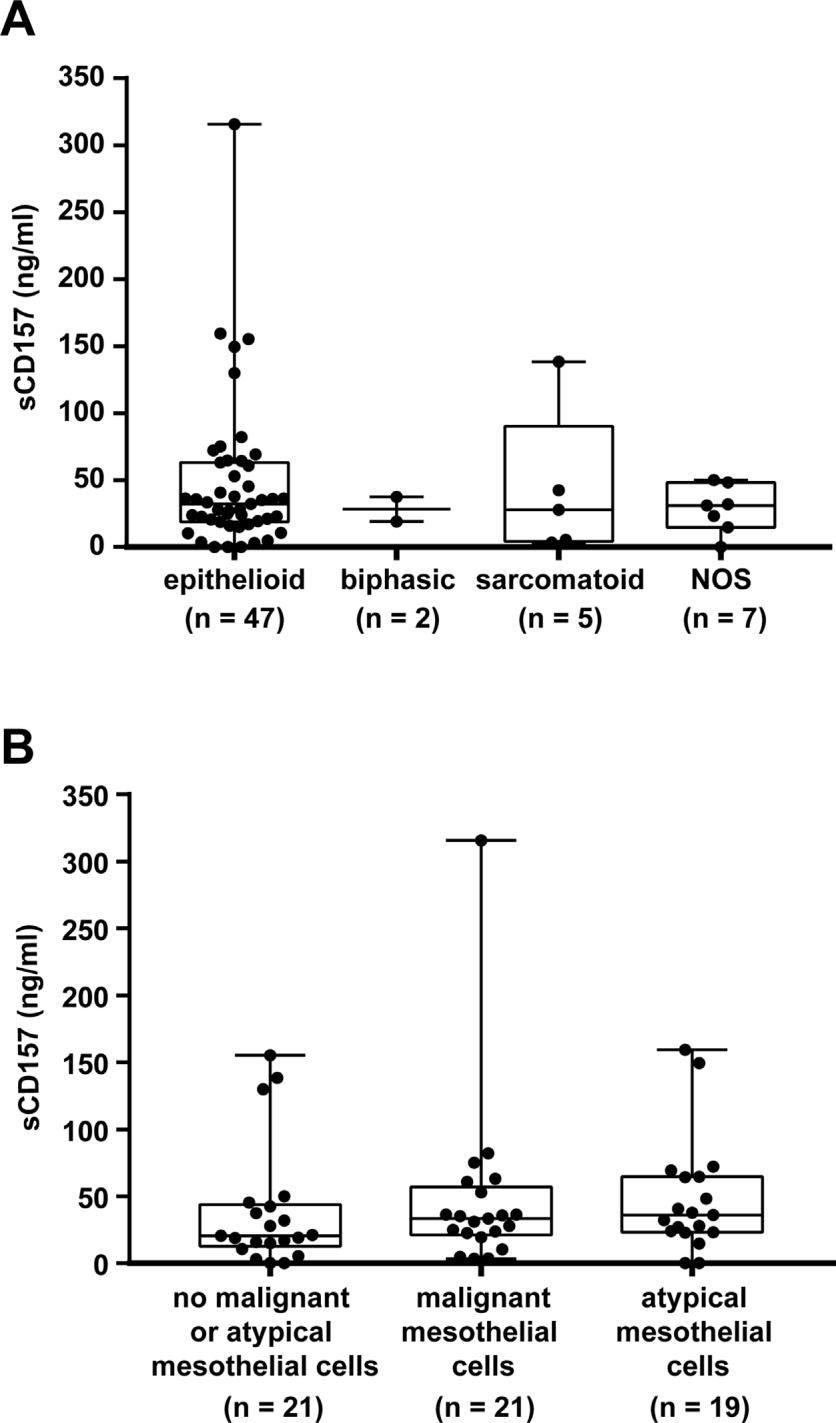
Analysis of MPM patients with high and low sCD157 levels in pleural effusion Quantification by the double determinant ELISA of sCD157 in pleural effusions from patients with MPM grouped in panel (**A**) according to the epithelioid, biphasic, sarcomatoid or NOS (not otherwise specified) histotype, and in panel (**B**) according to the presence or absence of neoplastic or atypical mesothelial cells. Each data point corresponds to sCD157 concentration of a single effusion sample, and represents the mean value of three independent experiments performed in duplicate. Boxes indicate the range (25th–75th percentiles), whiskers indicate major and minor values; the horizontal line within the boxes indicates the median sCD157 concentration of each group.

When the levels of sCD157 were compared in pleural effusions from patients with MPM of different histological type, no significant differences were observed (Figure 6A). However, due to the small number of patients with sarcomatoid and biphasic mesothelioma, this finding requires further analysis. Levels of sCD157 do not appear to be significantly influenced by the presence of malignant or atypical mesothelial cells in pleural effusions (Figure 6B) or by the presence of PMN (Table 2).

**Table 2:** Clinical and pathological characteristics of MPM patients and their association with sCD157 concentration in pleural effusions

	Cases	sCD157 <31.02 ng/ml	sCD157 ≥31.02 ng/ml	*P* value^*^
MPM, *n*	61	30	31	
Age at diagnosis, y				0.062
mean ± SD		73.07 ± 10,46	77.39 ± 6.76	
median (range)		73.50 (52–94)	79.00 (61–89)	
Sex				0.106
Male	40	23	17	
Female	21	7	14	
Histologic subtype				0.951
Epithelioid	47	23	24	
Sarcomatoid	5	3	2	
Biphasic	2	1	1	
NOS^a^	7	3	4	
Cytology				0.359
negative	21	13	8	
positive	21	9	12	
suspicious	19	8	11	
PMN^b^ (*n* = 54)				0.516
<10 cells × 10 (HPF, 200×)^c^	36	17	19	
10–50 cells × 1 (HPF, 200×)	14	8	6	
>50 cells × 10 (HPF, 200×)	4	1	3	
CD157 expression in biopsy tissues (*n* = 29)				0.06
H-score <110	11	8	3	
H-score ≥110	18	6	12	
Follow-up (*n* = 54)				0.005
DOD^d^	43	17	26	
AWD^e^	11	10	1	

^*^P values were determined using the Mann–Whitney *U*-test for independent samples, the two-sided χ^2^ or Fisher exact tests.

^a^NOS = Not otherwise specified.

^b^PMN = polymorphonuclear leukocytes.

^c^HPF = High Power Field, microscopy magnification 200×.

^d^DOD = died of disease.

^e^AWD = alive with disease.

Next we asked if there was a correlation between sCD157 levels in MPM effusions and CD157 expression in the primary tumor. We had 29 MPM biopsies for which residual specimens were available, and they were stained for CD157 by immunohistochemistry. CD157 was expressed in all 29 biopsy specimens examined at variable levels (median H-score = 110, IQR: 75–165) and with different (membrane/cytoplasmic) distribution patterns, as previously described [24]. When these patients were dichotomized according to the median sCD157 effusion concentration of the entire cohort (31.02 ng/ml, Table 1), 15 biopsies (51.72%) were from MPM patients with high sCD157 (≥31.02 ng/ml) in the corresponding effusion, and 14 (48.28%) from patients with low sCD157 (<31.02 ng/ml).

Next, tissue samples were grouped according to the median CD157 H-score to assess the relationship between CD157 expressed by the tumor and sCD157 in the effusion. Results showed a trend towards an association between CD157 staining and sCD157 in effusion of the same patient: 12/18 (66.67%) biopsies with a CD157 H-score ≥110 had high sCD157 concentrations in the corresponding pleural effusion compared to only 3/11 (16.67%) of those with a CD157 H-score < 110 (*P* = 0.06, Table 2).

At 1-year follow-up, data were available for 54 of the MPM patients in our cohort. At the time of testing, 27 patients had high effusion sCD157 (≥31.02 ng/ml) and 27 had low effusion sCD157 (<31.02 ng/ml). By the end of the observation period, 43 patients had died (median survival was 7 months) and 11 patients remained alive (median survival was 16 months). Of note, 26 of the 27 high sCD157 patients died of MPM in the follow-up period whereas only 17 of the 27 low sCD157 patients died (*P* = 0.005, Table 2).

### Soluble CD157 levels in pleural effusion and MPM patient survival

To test the possibility that sCD157 could be a prognostic marker, patients with MPM were grouped according to the median sCD157 concentration in effusions (31.02 ng/ml). The median survival of high sCD157 patients (≥31.02 ng/ml) was 10 months (95% CI = 1.519–18.481), while for low sCD157 patients the median survival was 16 months (95% CI = 6.654-25.346). Survival data for MPM patients with high sCD157 were plotted against survival data for patients with low sCD157 and tested for significant difference using the Kaplan-Meier method. Although the median survival was reduced in high sCD157 patients compared to low sCD157 patients, the difference in OS was not statistically significant (*P* = 0.574, log-rank), indicating that sCD157 effusion concentrations before diagnosis or at time of diagnosis was not a significant predictor of survival in MPM patients in this study.

## DISCUSSION

This study shows that CD157 is released *in vitro* both by malignant and non-malignant mesothelial cells and demonstrates the feasibility of measuring sCD157 *in vivo* in pleural effusions. Soluble CD157 levels are significantly higher in pleural effusions from MPM patients than in those from all other patient groups studied here. CD157 can be shed either as a soluble protein, generated by proteolytic cleavage of the membrane-bound protein, or as an exosome-anchored protein. Although not specific for MPM, exosomal CD157 has recently been identified among proteins that define a unique exosome-related signature in mesothelioma cell lines [32]. In view of CD157's pivotal role in cell-matrix interaction [19], it is tempting to speculate that exosomal CD157 in pleural effusions has the potential to participate to the cross-talk between tumor cells and the surrounding environment, thus influencing tumor behavior. In particular, MPM-derived exosomes have been shown to regulate cell adhesion and migration *in vitro* [33], both functions in which CD157 has a leading role in MPM [24] and ovarian cancer [23].

Pleural effusions form not only in MPM patients but also in other types of cancer and in a number of non-malignant pathologies. Thus, cytological examination is routinely adopted as the most common and safe diagnostic method to discriminate between malignant and non-malignant effusions. Nevertheless, despite high specificity, cytology often provides false negative results, even in repeated samplings, meaning that a positive result is informative, but a negative one is not. Moreover, there is a general consensus that the phenotypic discrimination of malignant mesothelial cells from non-malignant reactive cells is difficult, as is the distinction of MPM from other tumors, especially adenocarcinoma [34]. For these reasons, cytology is able to diagnose pleural metastases in 60–90% of tumors, but in only 30–40% of MPM [35] with higher sensitivity for epithelioid compared to sarcomatoid histotype [36]. Hence, the measurement of tumor markers in pleural effusions may represent a complementary tool for the diagnosis of MPM, or an alternative to thoracoscopy in subjects unfit for such an invasive procedure [37]. However, currently available markers offer insufficient sensitivity and specificity and are not used in clinical practice to diagnose MPM [38, 39].

Having detected sCD157 in pleural effusions, we explored its potential usefulness as a diagnostic tool. In our retrospective case series, sCD157 levels were significantly higher in MPM effusions than in all other pleural effusions, both non-MPM malignant and non-malignant. Instead, sCD157 levels were comparable in non-MPM malignant and non-malignant effusions, indicating that sCD157 is not a general marker of malignancy. Soluble CD157 was above the cut-off value of 23.66 ng/ml, indicating the best combination of sensitivity and specificity, in over 62% of effusions from MPM obtained before diagnosis of MPM was established, regardless the histological type and the identification of malignant or atypical mesothelial cells. Combined analysis of effusion sCD157 and biopsy CD157 from the same patient showed that higher levels of sCD157 tend to correlate with stronger immunohistochemical staining for CD157, suggesting that sCD157 derived at least in part from the tumor. We noted that four MPM effusions were virtually sCD157-negative, so it would seem that the absence of measurable amounts of sCD157 does not rule out the presence of MPM. Unfortunately, we could not test the biopsies from these four patients for CD157 expression as no material was available.

Why some MPM patients show little sCD157 in pleural effusions is currently under investigation. The finding that 26% of non-MPM malignant or non-malignant effusions had high sCD157 can probably be attributed, on the one hand, to expression of CD157 in certain tumors, such as peritoneal and ovarian cancers [23] (while no information on CD157 expression in breast cancer, lung cancer, kidney cancer and Hodgkin's lymphoma is so far available) and on the other, to the accumulation of proteases in effusions accompanying severe chronic inflammatory disorders. These proteases can cleave CD157 expressed in the stromal, endothelial [40] and inflammatory cells that form the microenvironment. Although the presence of PMN apparently does not influence the sCD157 levels in MPM effusions in our cohort, it is possible that in selected pathological contexts, inflammatory cells may release CD157. Indeed, high sCD157 was reported in sera from patients with rheumatoid arthritis, which is characterized by severe systemic chronic inflammatory conditions [13], and we also found high levels of sCD157 in two non-malignant effusions with elevated numbers of PMN.

Collectively, our data indicate that although sCD157 alone does not have the required accuracy for diagnostic purposes, measurement of effusion sCD157 can provide supporting evidence for diagnosing MPM in symptomatic individuals when cytology is inconclusive. Indeed, at 95.58% specificity, sCD157 evaluation in pleural effusions established a diagnosis of MPM in a subgroup of patients (9/40) undiagnosed by cytology (regardless of histotype), raising the sensitivity of effusion-based diagnosis of MPM from 34.42% (obtained by cytology) to 49.18%. Measuring sCD157 levels in pleural effusions is convenient as most patients routinely undergo thoracentesis to establish the nature of the effusion. Soluble CD157 can be assayed in stored samples, which allows clinicians to retrospectively request the test should cytology fail to establish a diagnosis.

Other biomarkers have been proposed for indirect diagnosis of MPM by pleural effusion, including mesothelin [41, 42] (the only biomarker approved by FDA as a humanitarian use device [43]), osteopontin [44], megakaryocyte potentiating factor (an alternative cleavage product of the mesothelin precursor protein) [45] and fibulin-3 [46], among others [47]. However, despite promising early results, none of these markers alone proved to have sufficient accuracy for MPM diagnosis [2, 48]. The inherent heterogeneity of MPM, emphasized by remarkable differences in phenotype and biological features among the various MPM subtypes [49], together with the lack of distinctive gene expression signatures [50] and key driver mutations [51, 52] make it unlikely that an ideal diagnostic biomarker for MPM will be found. Rather, it is plausible that combined analysis of a panel of biomarkers may be the right approach to ameliorate the clinical utility of effusion-based MPM diagnosis. This approach could have the advantage of shortening the time required for diagnosis and reduce the number of patients undergoing invasive procedures with the associated risks and costs. The next challenge will be to elucidate how to integrate dependable markers into effective diagnostic algorithms.

Our data indicate that, in this study, evaluation of sCD157 did not provide prognostic information for patients with MPM, even though the median survival of high sCD157 MPM patients (at diagnosis) was shorter than those of low sCD157 patients. Nevertheless, this result requires careful interpretation considering the small number of patients examined, and bearing in mind that the biphasic histotype, in which CD157 expression levels and prognosis are correlated [24] are under-represented in the analyzed case series.

This study was performed in a cohort of MPM patients from Northern Italy, which is considered a high-risk geographic area for mesothelioma because of asbestos mining and manufacturing in the past, before the ban came into effect [53]. We showed that detection of high levels of sCD157 may contribute to the diagnosis of an individual at risk of mesothelioma who presents with a pleural effusion unsuitable for the cytological diagnosis. However, for CD157 to confirm its potential clinical utility, independent diagnostic validations with separate geographic cohorts, representative of all mesothelioma histological types, should be performed. In addition, the potential usefulness of sCD157 as ‘risk factor’, or as marker of early diagnosis, should be evaluated in asbestos-exposed patients with benign effusions, which will require a prospective study. Another key step would be the combined evaluation of sCD157 and mesothelin in an attempt to improve diagnostics for all types of mesothelioma. It is noteworthy that 60% of effusions from sarcomatous mesothelioma showed high sCD157 concentrations, while mesothelin proved to be a useful marker for epithelioid mesothelioma but not for other histological variants [41, 42]. A concomitant analysis of the best-performing and reliable markers for virtually all mesothelioma histotypes may be the best approach to improve diagnosis and early detection of MPM in the near future.

## MATERIALS AND METHODS

### Cell lines

Met-5A non-malignant, SV40-immortalized pleural mesothelial cells were purchased from American Type Culture Collection (ATCC, Manassas, VA). MPP89 and MSTO-211H mesothelioma cells were obtained from Interlab Cell Line Collection (Advanced Biotechnology Center, Genova, Italy). REN and MPP cells were kindly provided by L. Moro (University of Novara, Italy) who received the cells from S. Albelda (University of Pennsylvania, Philadelphia, USA). CG98 and MM98 MPM cells were obtained from the mesothelioma bio-bank at the Pathology Unit, City Hospital of Alessandria, Italy [54]. All cells were thawed from early-passage frozen stocks and were passaged less than six times prior to use. Cells were regularly examined for absence of *Mycoplasma* contamination by a PCR-based assay.

### Immunofluorescence and flow cytometry

Cells (2 × 10^5^) were incubated for 30 min at 4°C with SY/11B5 anti-CD157 mAb (5 μg/ml) [23], washed and incubated for 30 min at 4°C with F(ab')_2_-goat-anti-mouse IgG-FITC (Jackson ImmunoResearch, DBA, Milano, Italy). Flow cytometry was carried out using a BD FACS Canto I (BD Biosciences, Milano, Italy) and analysed by FlowJo^®^ software.

### Western blot analysis

MPM cells were grown to 80% confluence in culture medium containing 10% fetal calf serum (FCS), then maintained in FCS-free conditions. At the indicated time, both cells and culture medium were collected and processed. The culture medium was centrifuged for 10 min at 300 ***×*** *g* and 20 min at 10,000 × *g*, to remove residual cells and debris. Proteins were precipitated with trichloroacetic acid (TCA), washed twice with acetone, air-dried and dissolved in 2× non-reducing SDS sample buffer (30% sucrose, 80 mM Tris-HCl pH 8.8, 3% SDS, 0.01 mg/ml bromophenol blue). Total lysates were obtained in ice-cold RIPA lysis buffer supplemented with protease inhibitor cocktail (Sigma-Aldrich). Protein concentration was determined by the Quick Start Bradford protein assay (Bio-Rad, Milano, Italy) using bovine serum albumin as standard. Equal amounts of proteins (30 μg/lane) were separated by SDS-PAGE, transferred to polyvinylidene difluoride (PVDF) membranes, blocked with 5% milk, and probed with the indicated primary antibody, followed by horseradish peroxidase (HRP)-conjugated goat-anti-mouse polyclonal antibody and HRP-conjugated anti-β-Actin antibody (Santa Cruz Biotechnologies, Milano, Italy). Immunoreactive bands were visualized by Westar ECL substrate (Bio-Rad). Images were captured with a ChemiDoc™ XRS+ System.

### Isolation of microvesicles and exosomes from cell culture medium

Vesicles were isolated from culture medium of cells maintained in FCS-free medium for the indicated time, as described [55]. FCS-free culture medium was collected and centrifuged for 10 min at 300 × *g* to remove residual cells, then supernatants were centrifuged for 20 min at 2,000 × *g* and for 30 min at 10,000 × *g*, to remove debris. Membrane vesicles were centrifuged at 100,000 × *g* for 2 h, washed in PBS, and ultracentrifuged at 100,000 × *g* for 1 h. For Western blotting, vesicles were suspended in RIPA buffer and quantified using the Bradford method. Equal amounts of protein were boiled with non-reducing SDS-sample buffer and further processed.

To isolate exosomes, vesicles were suspended in 0.25 M sucrose in TBS and loaded onto a 5-step gradient comprising layers of 2, 1.3, 1.16, 0.8 and 0.5 M sucrose in TBS, as described [56] and centrifuged at 100,000 × *g* for 2.5 h. Twelve fractions (400 μl/each) were collected from the top of the gradient and proteins in each fraction were precipitated with methanol/chloroform and analyzed by SDS-PAGE and Western blot.

### Isolation of exosomes from pleural effusions

Exosomes from pleural effusions were isolated using the Total Exosomes Isolation kit (Life Technologies, Monza, Italy) following the manufacturer's instructions. Briefly, 200 μl of pleural effusion were centrifuged at 2,000 × *g*, at room temperature for 30 min to remove cells and debris. Then the appropriate amount of Total Exosomes Isolation reagent was added, according to the manufacturer's recommendations. Mixtures were vortexed and incubated at room temperature for 30 min before centrifugation at 10,000 × g for 10 min to collect exosomes. Pellets containing exosomes were suspended in RIPA buffer and quantified using the Bradford method. Selected effusion samples were processed for exosome purification by sucrose density gradient, as described above, and analyzed by SDS-PAGE and Western blot.

### Enzyme-linked immunosorbent assay (ELISA)

sCD157 levels were quantified in ng/ml using a double determinant ELISA. MaxiSorp microtiter plates (Nunc, Roskilde, Denmark) were coated overnight at 4°C with 5 μg/ml of SY/11B5 anti-CD157 mAb in PBS. After blocking with Pierce™ Protein-Free T20 Blocking Buffer (PFBB-T, Pierce, Thermo Fisher Scientific, Milano, Italy) for 2 h at room temperature, samples (100 μl/well) were added and incubated overnight at 4°C. Cell culture medium was used undiluted and pleural effusions were diluted 1:50 in PFBB-T. All samples were analysed in duplicate in at least three independent tests. After four washes with PFBB-T (300 μl/well), 0.5 μg/ml of rabbit polyclonal IgG to human CD157 (Sigma-Aldrich) were added for 2 h at room temperature. After four washes with PFBB-T, plates were incubated for 1 h at 37°C with HRP-conjugated anti-rabbit IgG (1:5,000 in PFBB-T). After extensive washing, the reaction was developed with 100 μl of tetramethylbenzidine (TMB; BioFX, Milano, Italy) for 10 min at room temperature in the dark, then stopped with 50 μl of 0.5 M H_2_SO_4_. A nine-point standard curve (concentration range 50 to 0.195 ng/ml) was obtained using recombinant, human His-tagged CD157 (rhCD157, R&D System, Milano, Italy). Absorbance read at 450 nm was used to determine sCD157 concentration by comparison of means of the replicate measurements with the standard curve performed in each plate. The standard curve was generated by plotting the mean O.D. for each standard on the y-axis and the concentration on the x-axis and drawing a best fit curve through the points on the graph using a four parameter logistic (4-PL) curve fit. To validate the accuracy of our results, the intra-assay and inter-assay coefficient of variation (CV) were determined. Three effusion samples with low, middle and high levels of sCD157 were tested ten times on one plate (precision within an assay), or in three different plates, 6 replicates in each plate, (precision between assays). We obtained an intra- and inter-assay CV <9% and <7%, respectively (not shown).

### Study participants

Pleural effusions (2 ml/each) were obtained from 295 consecutive patients who underwent thoracentesis at the Division of Pathology, Thoracic Oncology and Thoracic Surgery at San Luigi Gonzaga Hospital, Torino, Italy, between November 2012 and October 2016. The diagnosis of MPM was based on clinical signs, imaging data, cytological examination of pleural effusions and/or histology of pleural biopsies. Patients with MPM were enrolled before starting any therapeutic treatment. Fresh effusion samples were retrieved without anticoagulant, centrifuged for 10 min at 2,000 × *g*, and immediately stored at –80°C until use. All specimens were analysed by cytology and, when necessary for diagnosis, by immunohistochemistry of pleural biopsies taken during thoracoscopy. Effusions were considered as malignant based on a demonstration of malignant cells by cytology and/or by histology in biopsy specimens. Patients were defined as having non-malignant diseases when there were no signs of ongoing or previous tumor and a one year of follow-up did not show any clinical evidence of tumor. No information was available concerning exposure to asbestos, and no attempt was made to distinguish exudates from transudates. Metastatic pleural diseases correlated with advanced stage, while information of stage for MPM patients was not available for this study. All assays were performed on coded samples by technical staff unaware of patient's diagnosis. The study was approved by the San Luigi Gonzaga Hospital Institutional Review Board and participants gave informed written consent. All studies on human subjects were carried out according to the Declaration of Helsinki.

### Immunohistochemistry

Sections (5 μm) from formalin-fixed MPM thoracoscopic biopsies were analysed for CD157 expression using the RF3 anti-CD157 mAb (Space, Milano, Italy), as described [24]. CD157 expression was evaluated using a semi-quantitative histological score (H-score) calculated by summing the percentage of cells stained at each intensity level (0–100) in the whole section, multiplied by the weighted intensity (0, none; 1, weak; 2, moderate; 3, intense), generating for each tumor a score ranging from 0 to 300. An experienced pathologist, blind of the levels of sCD157 in effusions, evaluated all slides.

### Statistical analysis

Unless otherwise indicated, results are expressed as mean value ± s.e.m. Comparison between groups were performed using the non-parametric Mann-Whitney *U*-test for independent samples, with adjustment for multiple comparisons using the Dunn's method. sCD157 levels were shown as median and interquartile range (IQR: 25th and 75th percentiles). Means, SD, median, and range were used to describe the age of patients; other clinical and pathological characteristics of MPM patients were shown in terms of number of cases and grouped according to the median sCD157 concentration (<31.02 or ≥31.02 ng/ml). The association between sCD157 concentration and clinical data was analysed using the ***χ***^2^ test or Fisher exact test. The distribution of sCD157 values among the groups under study was evaluated by receiver operating characteristics (ROC) analysis, using the area under the ROC curve (AUC) as a measure of accuracy. AUC were reported with their 95% confidence intervals (95% CI). On the basis of the ROC analysis, the optimal sCD157 cut-off point of discrimination between the study subgroups was determined using the Youden Index, which reflects the joint maximum values of sensitivity and specificity. Survival time was defined as time between pleural effusion withdrawal and date of death or date of last follow-up. Patients still alive at last follow-up were considered censored. Patients alive but not reaching at least median OS (11 months) were excluded from this analysis. The Kaplan-Meier method was used for survival estimation and the log-rank test was performed to compare differences in OS between groups. A *P* value < 0.05 was considered statistically significant. Statistical analyses were performed using GraphPad Prism 6.01 and SPSS statistics V.24.

## References

[1] Roe OD, Stella GM (2015). Malignant pleural mesothelioma: history, controversy and future of a manmade epidemic. Eur Respir Rev.

[2] Bibby AC, Tsim S, Kanellakis N, Ball H, Talbot DC, Blyth KG, Maskell NA, Psallidas I (2016). Malignant pleural mesothelioma: an update on investigation, diagnosis and treatment. Eur Respir Rev.

[3] van Meerbeeck JP, Gaafar R, Manegold C, Van Klaveren RJ, Van Marck EA, Vincent M, Legrand C, Bottomley A, Debruyne C, Giaccone G, European Organisation for R, Treatment of Cancer Lung Cancer G, National Cancer Institute of C (2005). Randomized phase III study of cisplatin with or without raltitrexed in patients with malignant pleural mesothelioma: an intergroup study of the European Organisation for Research and Treatment of Cancer Lung Cancer Group and the National Cancer Institute of Canada. J Clin Oncol.

[4] Rudd RM (2010). Malignant mesothelioma. Br Med Bull.

[5] Segal A, Sterrett GF, Frost FA, Shilkin KB, Olsen NJ, Musk AW, Nowak AK, Robinson BW, Creaney J (2013). A diagnosis of malignant pleural mesothelioma can be made by effusion cytology: results of a 20 year audit. Pathology.

[6] Canessa PA, Ferro P, Manta C, Sivori M, Franceschini MC, Fedeli F, Roncella S (2013). Clinical value of mesothelin in pleural effusions versus histology by medical thoracoscopy: brief report. Med Oncol.

[7] Lagniau S, Lamote K, van Meerbeeck JP, Vermaelen KY (2017). Biomarkers for early diagnosis of malignant mesothelioma: Do we need another moonshot?. Oncotarget.

[8] Ferrero E, Saccucci F, Malavasi F (1999). The human CD38 gene: polymorphism, CpG island, and linkage to the CD157 (BST-1) gene. Immunogenetics.

[9] Malavasi F, Deaglio S, Funaro A, Ferrero E, Horenstein AL, Ortolan E, Vaisitti T, Aydin S (2008). Evolution and function of the ADP ribosyl cyclase/CD38 gene family in physiology and pathology. Physiol Rev.

[10] Ferrero E, Lo Buono N, Horenstein AL, Funaro A, Malavasi F (2014). The ADP-ribosyl cyclases—the current evolutionary state of the ARCs. Front Biosci (Landmark Ed).

[11] Ferrero E, Lo Buono N, Morone S, Parrotta R, Mancini C, Brusco A, Giacomino A, Augeri S, Rosal-Vela A, Garcia-Rodriguez S, Zubiaur M, Sancho J, Fiorio Pla A (2017). Human canonical CD157/Bst1 is an alternatively spliced isoform masking a previously unidentified primate-specific exon included in a novel transcript. Sci Rep.

[12] Funaro A, Ortolan E, Bovino P, Lo Buono N, Nacci G, Parrotta R, Ferrero E, Malavasi F (2009). Ectoenzymes and innate immunity: the role of human CD157 in leukocyte trafficking. Front Biosci (Landmark Ed).

[13] Lee BO, Ishihara K, Denno K, Kobune Y, Itoh M, Muraoka O, Kaisho T, Sasaki T, Ochi T, Hirano T (1996). Elevated levels of the soluble form of bone marrow stromal cell antigen 1 in the sera of patients with severe rheumatoid arthritis. Arthritis Rheum.

[14] Funaro A, Ortolan E, Ferranti B, Gargiulo L, Notaro R, Luzzatto L, Malavasi F (2004). CD157 is an important mediator of neutrophil adhesion and migration. Blood.

[15] Ortolan E, Vacca P, Capobianco A, Armando E, Crivellin F, Horenstein A, Malavasi F (2002). CD157, the Janus of CD38 but with a unique personality. Cell Biochem Funct.

[16] Ortolan E, Tibaldi EV, Ferranti B, Lavagno L, Garbarino G, Notaro R, Luzzatto L, Malavasi F, Funaro A (2006). CD157 plays a pivotal role in neutrophil transendothelial migration. Blood.

[17] Kaisho T, Ishikawa J, Oritani K, Inazawa J, Tomizawa H, Muraoka O, Ochi T, Hirano T (1994). BST-1, a surface molecule of bone marrow stromal cell lines that facilitates pre-B-cell growth. Proc Natl Acad Sci U S A.

[18] Krupka C, Lichtenegger FS, Kohnke T, Bogeholz J, Bucklein V, Roiss M, Altmann T, Do TU, Dusek R, Wilson K, Bisht A, Terrett J, Aud D (2017). Targeting CD157 in AML using a novel, Fc-engineered antibody construct. Oncotarget.

[19] Morone S, Augeri S, Cuccioloni M, Mozzicafreddo M, Angeletti M, Lo Buono N, Giacomino A, Ortolan E, Funaro A (2014). Binding of CD157 protein to fibronectin regulates cell adhesion and spreading. J Biol Chem.

[20] Lo Buono N, Morone S, Giacomino A, Parrotta R, Ferrero E, Malavasi F, Ortolan E, Funaro A (2014). CD157 at the intersection between leukocyte trafficking and epithelial ovarian cancer invasion. Front Biosci (Landmark Ed).

[21] Quarona V, Zaccarello G, Chillemi A, Brunetti E, Singh VK, Ferrero E, Funaro A, Horenstein AL, Malavasi F (2013). CD38 and CD157: A long journey from activation markers to multifunctional molecules. Cytometry B Clin Cytom.

[22] Morone S, Lo Buono N, Parrotta R, Giacomino A, Nacci G, Brusco A, Larionov A, Ostano A, Mello-Grand M, Chiorino G, Ortolan E, Funaro A (2012). Overexpression of CD157 contributes to epithelial ovarian cancer progression by promoting mesenchymal differentiation. PLoS One.

[23] Ortolan E, Arisio R, Morone S, Bovino P, Lo-Buono N, Nacci G, Parrotta R, Katsaros D, Rapa I, Migliaretti G, Ferrero E, Volante M, Funaro A (2010). Functional Role and prognostic significance of CD157 in ovarian carcinoma. J Natl Cancer Inst.

[24] Ortolan E, Giacomino A, Martinetto F, Morone S, Lo Buono N, Ferrero E, Scagliotti G, Novello S, Orecchia S, Ruffini E, Rapa I, Righi L, Volante M, Funaro A (2014). CD157 enhances malignant pleural mesothelioma aggressiveness and predicts poor clinical outcome. Oncotarget.

[25] Yamamoto-Katayama S, Sato A, Ariyoshi M, Suyama M, Ishihara K, Hirano T, Nakamura H, Morikawa K, Jingami H (2001). Site-directed removal of N-glycosylation sites in BST-1/CD157: effects on molecular and functional heterogeneity. Biochem J.

[26] Lo Buono N, Parrotta R, Morone S, Bovino P, Nacci G, Ortolan E, Horenstein AL, Inzhutova A, Ferrero E, Funaro A (2011). The CD157-integrin partnership controls transendothelial migration and adhesion of human monocytes. J Biol Chem.

[27] Kowal J, Arras G, Colombo M, Jouve M, Morath JP, Primdal-Bengtson B, Dingli F, Loew D, Tkach M, Thery C (2016). Proteomic comparison defines novel markers to characterize heterogeneous populations of extracellular vesicle subtypes. Proc Natl Acad Sci U S A.

[28] de Gassart A, Geminard C, Fevrier B, Raposo G, Vidal M (2003). Lipid raft-associated protein sorting in exosomes. Blood.

[29] Colombo M, Raposo G, Thery C (2014). Biogenesis, secretion, and intercellular interactions of exosomes and other extracellular vesicles. Annu Rev Cell Dev Biol.

[30] Xu R, Greening DW, Zhu HJ, Takahashi N, Simpson RJ (2016). Extracellular vesicle isolation and characterization: toward clinical application. J Clin Invest.

[31] Li M, Rai AJ, DeCastro GJ, Zeringer E, Barta T, Magdaleno S, Setterquist R, Vlassov AV (2015). An optimized procedure for exosome isolation and analysis using serum samples: Application to cancer biomarker discovery. Methods.

[32] Greening DW, Ji H, Chen M, Robinson BW, Dick IM, Creaney J, Simpson RJ (2016). Secreted primary human malignant mesothelioma exosome signature reflects oncogenic cargo. Sci Rep.

[33] Hegmans JP, Bard MP, Hemmes A, Luider TM, Kleijmeer MJ, Prins JB, Zitvogel L, Burgers SA, Hoogsteden HC, Lambrecht BN (2004). Proteomic analysis of exosomes secreted by human mesothelioma cells. Am J Pathol.

[34] Husain AN, Colby T, Ordonez N, Krausz T, Attanoos R, Beasley MB, Borczuk AC, Butnor K, Cagle PT, Chirieac LR, Churg A, Dacic S, Fraire A (2013). Guidelines for pathologic diagnosis of malignant mesothelioma: 2012 update of the consensus statement from the International Mesothelioma Interest Group. Arch Pathol Lab Med.

[35] Henderson DW, Reid G, Kao SC, van Zandwijk N, Klebe S (2013). Challenges and controversies in the diagnosis of malignant mesothelioma: Part 2. Malignant mesothelioma subtypes, pleural synovial sarcoma, molecular and prognostic aspects of mesothelioma, BAP1, aquaporin-1 and microRNA. J Clin Pathol.

[36] Rakha EA, Patil S, Abdulla K, Abdulkader M, Chaudry Z, Soomro IN (2010). The sensitivity of cytologic evaluation of pleural fluid in the diagnosis of malignant mesothelioma. Diagn Cytopathol.

[37] Pass HI, Carbone M (2009). Current status of screening for malignant pleural mesothelioma. Semin Thorac Cardiovasc Surg.

[38] Arnold DT, De Fonseka D, Hamilton FW, Rahman NM, Maskell NA (2017). Prognostication and monitoring of mesothelioma using biomarkers: a systematic review. Br J Cancer.

[39] Chen Z, Gaudino G, Pass HI, Carbone M, Yang H (2017). Diagnostic and prognostic biomarkers for malignant mesothelioma: an update. Transl Lung Cancer Res.

[40] Smirnov DA, Foulk BW, Doyle GV, Connelly MC, Terstappen LW, O’Hara SM (2006). Global gene expression profiling of circulating endothelial cells in patients with metastatic carcinomas. Cancer Res.

[41] Robinson BW, Creaney J, Lake R, Nowak A, Musk AW, de Klerk N, Winzell P, Hellstrom KE, Hellstrom I (2003). Mesothelin-family proteins and diagnosis of mesothelioma. Lancet.

[42] Creaney J, Yeoman D, Naumoff LK, Hof M, Segal A, Musk AW, De Klerk N, Horick N, Skates SJ, Robinson BW (2007). Soluble mesothelin in effusions: a useful tool for the diagnosis of malignant mesothelioma. Thorax.

[43] Li ZQ, Verch T, Allard WJ (2007). MESOMARK((R)) in vitro diagnostic test for mesothelioma. Expert Opin Med Diagn.

[44] Grigoriu BD, Scherpereel A, Devos P, Chahine B, Letourneux M, Lebailly P, Gregoire M, Porte H, Copin MC, Lassalle P (2007). Utility of osteopontin and serum mesothelin in malignant pleural mesothelioma diagnosis and prognosis assessment. Clin Cancer Res.

[45] Hollevoet K, Nackaerts K, Gosselin R, De Wever W, Bosquee L, De Vuyst P, Germonpre P, Kellen E, Legrand C, Kishi Y, Delanghe JR, van Meerbeeck JP (2011). Soluble mesothelin, megakaryocyte potentiating factor, and osteopontin as markers of patient response and outcome in mesothelioma. J Thorac Oncol.

[46] Pass HI, Levin SM, Harbut MR, Melamed J, Chiriboga L, Donington J, Huflejt M, Carbone M, Chia D, Goodglick L, Goodman GE, Thornquist MD, Liu G (2012). Fibulin-3 as a blood and effusion biomarker for pleural mesothelioma. N Engl J Med.

[47] Sun HH, Vaynblat A, Pass HI (2017). Diagnosis and prognosis-review of biomarkers for mesothelioma. Ann Transl Med.

[48] Creaney J, Robinson BWS (2017). Malignant Mesothelioma Biomarkers: From Discovery to Use in Clinical Practice for Diagnosis, Monitoring, Screening, and Treatment. Chest.

[49] Yap TA, Aerts JG, Popat S, Fennell DA (2017). Novel insights into mesothelioma biology and implications for therapy. Nat Rev Cancer.

[50] Gordon GJ (2005). Transcriptional profiling of mesothelioma using microarrays. Lung Cancer.

[51] Kiyotani K, Park JH, Inoue H, Husain A, Olugbile S, Zewde M, Nakamura Y, Vigneswaran WT (2017). Integrated analysis of somatic mutations and immune microenvironment in malignant pleural mesothelioma. Oncoimmunology.

[52] Guo G, Chmielecki J, Goparaju C, Heguy A, Dolgalev I, Carbone M, Seepo S, Meyerson M, Pass HI (2015). Whole-exome sequencing reveals frequent genetic alterations in BAP1, NF2, CDKN2A, and CUL1 in malignant pleural mesothelioma. Cancer Res.

[53] Corfiati M, Scarselli A, Binazzi A, Di Marzio D, Verardo M, Mirabelli D, Gennaro V, Mensi C, Schallemberg G, Merler E, Negro C, Romanelli A, Chellini E (2015). Epidemiological patterns of asbestos exposure and spatial clusters of incident cases of malignant mesothelioma from the Italian national registry. BMC Cancer.

[54] Orecchia S, Schillaci F, Salvio M, Libener R, Betta PG (2004). Aberrant E-cadherin and gamma-catenin expression in malignant mesothelioma and its diagnostic and biological relevance. Lung Cancer.

[55] Thery C, Amigorena S, Raposo G, Clayton A (2006). Isolation and characterization of exosomes from cell culture supernatants and biological fluids. Curr Protoc Cell Biol.

[56] Gutwein P, Stoeck A, Riedle S, Gast D, Runz S, Condon TP, Marme A, Phong MC, Linderkamp O, Skorokhod A, Altevogt P (2005). Cleavage of L1 in exosomes and apoptotic membrane vesicles released from ovarian carcinoma cells. Clin Cancer Res.

